# Cloth sharing with a scabies case considerably explains human scabies among children in a low socioeconomic rural community of Ethiopia

**DOI:** 10.1186/s41182-023-00544-6

**Published:** 2023-09-15

**Authors:** Fekadie Melese, Asmamaw Malede, Tadesse Sisay, Abraham Geremew, Mesfin Gebrehiwot, Lebasie Woretaw, Getu Atanaw, Jember Azanaw, Mihret Melese, Hailemariam Feleke

**Affiliations:** 1 Hospital Quality Unit and Public Health Emergency Management Office, Nefas Mewcha Primary Hospital, Nefas Mewcha, Ethiopia; 2 Department of Environmental Health, Wollo University, Dessie, Ethiopia; 3 Department of Environmental Health, Haramaya University, Harar, Ethiopia; 4 Department of Obstetrics and Gynecology, University of Gondar, Gondar, Ethiopia; 5 Department of Environmental and Occupational Health and Safety, University of Gondar, Gondar, Ethiopia; 6 Department of Medical Physiology, University of Gondar, Gondar, Ethiopia

**Keywords:** Scabies infestation, Poor socioeconomy, Unsafe water supply, Amhara region

## Abstract

**Background:**

In 2020, scabies were integrated into the WHO roadmap for neglected tropical diseases, aimed at ending the negligence to realize the SDGs. Ethiopia has also introduced scabies as a notifiable disease in drought-prone localities since 2015. Many of the previous studies employed study designs that might be subject to bias. Moreover, there is no scientific evidence about scabies in this area. Hence, this study aimed to determine the prevalence and associated factors of scabies among children aged below 15 years in rural Ethiopia.

**Methods:**

A community-based cross-sectional study was carried out among 942 children in rural *kebeles* of *Lay Gayent District* from March through May 15, 2021. A two-stage sampling technique was applied. Data on sociodemographics, housing, water supply and sanitation, children’s personal hygiene, and caregivers’ knowledge about scabies were collected by a structured questionnaire. Data quality was maintained through pretesting, training of data collectors and supervisors, and supervision. An adjusted binary logistic regression was modelled to identify factors associated with scabies. The Hosmer–Lemeshow goodness-of-fit test was run to check the model fitness.

**Results:**

The prevalence of scabies was 21.5% (95% CI 19.0–24.3). Maternal illiteracy (adjusted odds ratio (AOR) = 1.61; 95% CI 1.07–2.43); low household wealth (AOR = 2.04; 95% CI 1.25–3.33); unimproved water source (AOR = 1.58; 95% CI 1.05–2.40); not cleaning a house daily (AOR = 2.43; 95% CI 1.63–3.62); not trimming nails (AOR = 2.21; 95% CI 1.50–3.25); cloth sharing with a scabies case (AOR = 11.77; 95% CI 6.94–19.97); and low caregiver knowledge about scabies (AOR = 2.44; 95% CI 1.64–3.63) were factors associated with scabies.

**Conclusions:**

Scabies remains a significant public health issue among children aged below 15 years in the district. Maternal illiteracy, low household wealth, unimproved water source, not cleaning a house daily, not trimming nails, cloth sharing with a scabies case, and low caregiver knowledge about scabies were factors associated with scabies. Community-wide interventions with prime emphasis on improving maternal education and caregivers’ knowledge about scabies, upgrading household wealth, ensuring a safe water supply, providing healthy housing, and ensuring adequate personal hygiene are warranted.

## Background

Human scabies has a worldwide distribution and remains one of the most common, easily treatable dermal diseases in developing countries, mainly in resource-poor communities [1, 2]. It has a substantial impact via clinical management cost, work or school absenteeism and psychosocial consequences [3–6]. Skin manifestations, such as papules, burrows and intense itching, occur due to host defense mechanisms for itch mite products [7]. Commonly, transmission occurs among household (HH) members, indiscriminate sexual partners, and within institutions [8]. Ample evidence is documented on the high prevalence of scabies resulting from poverty and overcrowding, primarily within sleeping accommodations [9–11].

Scabies is a skin disease that causes considerable disability-adjusted life-years [12]. Globally, its prevalence ranges from 0.2% to 71% [13], and the burden is high in tropical and humid regions, primarily in children [14]. In developing countries, the average prevalence in children is estimated to be 5 to 10% [15]. Elsewhere in the world, human scabies prevalence rates of 25.7% in the Solomon Islands [16], 5.2% in the dry season and 1.9% in the wet season in Guinea-Bissau [17], and 3.1% in southeastern Iran [18] among children have been reported. Recently, large outbreaks have been reported in Africa, including Ethiopia, which screened more than one million persons [3]. In Ethiopia, the disease is principally common in rural poor and hard-to-reach communities. The pooled prevalence of the disease among children was 19.5% [19]. In the Amhara Region, a study performed in drought-affected areas indicated a high prevalence of the disease in school-aged children, accounting for nearly half (49%) of the cases [3]. In addition, a community-based survey in the Amhara Region, northwest Ethiopia, reported a 10.8% prevalence of scabies among school-age children [20].

The main predisposing factors for scabies infestations are poverty or low socioeconomic status [10, 18, 21–23], poor personal and domestic hygiene [22–24], sharing of fomites [10, 11, 18, 21], and overcrowded conditions [9, 10, 23]. In 2020, scabies were integrated into the WHO roadmap for neglected tropical diseases (NTDs) 2021–2030, aimed at ending the negligence to realize the Sustainable Development Goals [25]. Ethiopia has also introduced scabies as a notifiable disease in localities prone to drought since 2015 [3, 26].

Unlike the Pacific region or Latin America [13], robust epidemiological surveys for scabies have not been undertaken in the East African region, including Ethiopia. Despite having a high burden of morbidity related to scabies, top-quality epidemiological information on its prevalence and the associated factors in Ethiopia is lacking. Many of the previous studies employed study designs that might be subject to bias such as school-based surveys or involving persons who claim a dermal problem [4, 27]. Since scabies may result in stigma and poor school attendance, these research designs may distort the true prevalence of scabies. Moreover, there is no evidence about scabies prevalence and its associated factors among rural children in the study area.

To address this gap, this study was conducted to determine the prevalence of human scabies and its associated factors among children under 15 years of age in rural areas of Ethiopia. Therefore, the findings may assist in directing proper implementation of scabies-specific interventions to significantly reduce the burden of the disease in Ethiopia.

## Methods

### Study design, period and area

A community-based cross-sectional study was carried out in 26 rural *kebeles* (the lowest administrative units in Ethiopia) of *Lay Gayent District*, northwest Ethiopia, from March 1 to May 15, 2021. The *district* had five urban *kebeles*. It is characterized by four temperature-based agro-ecological zones: lowlands, midlands, highlands, and alpine. In the *district*, the main rainy season occurs between June and September, and the minor rainy season occurs between March and May. In 2020/2021, the district had a projected population of 248,338 (122,646 were males and 125,692 were females) residents [28] and 46,038 HHs. In the projected population, children aged below 15 years accounted for 105,876 persons. It had nine health centers, thirty health posts and one *district* hospital.

### Source population and eligibility criteria

The source population for this study was mothers/caregivers-children aged below 15 years residing in rural *kebeles* of Lay Gayent *District*. Children aged 1 to 14 years who had been permanent dwellers in the rural *kebeles* of the district were eligible. Children who had other dermal illnesses or severe diseases were excluded from this study.

### Sample size computation

Sample size was computed using the single population proportion formula [29] with the following assumptions: 95% confidence level (Z_α/2_= 1.96), *p* was an estimate of the prevalence of scabies infestation among children aged below 15 years (23.8%) [30], and *d* degree of precision or margin of error (4%):

$$n =\frac{{({Z}_{{\alpha /}_{2}})}^{2}* p (1-p)}{{d}^{2}}$$

In addition, due to the nature of the sampling scheme, which is a two-stage sampling technique, the design effect (Deff = 2) was considered to maintain the variability in the population through the increment of the variance between the clusters. The lowered precision due to the noninvolvement of all clusters can be minimized by considering the design effect [31]. Moreover, a 10% contingency was considered for both the estimated sample size and the nonrespondents, which was calculated by (final sample size = $$\left( {\text{final sample size}\, = {\raise0.7ex\hbox{$n$} \!\mathord{\left/ {\vphantom {n {1 - 10\% }}}\right. \kern-\nulldelimiterspace} \!\lower0.7ex\hbox{${1 - 10\% }$}}} \right)$$). Thus, the final sample size was determined to be 967.

### Study population and sampling technique

A two-stage sampling technique was used to select the study subjects. First, nine rural *kebeles* (> 30% of the total rural *kebeles*) were selected using the lottery method from the 26 rural *kebeles* of the *district*. A total number and list of HHs that had children aged below 15 years in selected *kebeles* were taken from the family folder record of health posts. Then, the sample size was proportionally allocated for the selected *kebeles* based on the number of HHs that had children aged below 15 years in each *kebele*. Second, HHs were selected by simple random sampling. For HHs who had more than one child aged below 15 years, one child was selected randomly. When study subjects (child aged below 15 years-mother–father) were not present in an HH at the first visit, the HH was revisited once more on the same day or the next day. If a study subject was not present at the third visit, the subject was entertained as a nonrespondent.

### Data collection and quality control

Scabies infestation of children was diagnosed by the Delphi method, which is a standardized and accepted diagnostic method [32]. A child was confirmed as a scabies case when he/she had clinical signs and symptoms of scabies and the presence of mites, eggs or faeces on light microscopy of skin samples (Level A1) [32, 33]. Two medical laboratory technologists who are certified by the Ethiopian Federal Ministry of Health diagnosed children for having scabies infestation via light microscopy. Scabies diagnosis training for medical laboratory technologists was given for 2 days. A sterile scalpel blade was used to scrape the skin sideways over an area of a suspected burrow or lesion (covered with a drop of mineral oil), and the skin scrape sample was taken and placed on a glass slide that had mineral oil on its center. A glass slide that contained a skin scrape sample was labelled with a participant identification number to link with the respective questionnaire data. Then, skin scraping samples were placed in a slide carrier and transported to *Zagoch* Health Center or *Nefas Mewcha* Primary Hospital for definitive diagnosis.

Sociodemographic, socioeconomic, housing, water supply and sanitation, child personal hygiene, and caregiver knowledge about scabies characteristics were collected by a structured questionnaire. The knowledge tool included 21 questions about scabies. A point was marked for each correct response, and a score of zero was given for wrong or uncertain responses. All questions had a total of 21 points. The survey data were collected through face-to-face interviews with mothers, fathers, or caregivers and/or direct observation by five diploma holder nurses. Two BSc holder Environmental Health professionals supervised the data collection. Data collectors and supervisors were trained on how to collect the survey data for 3 days. Regular supervision was set up by the trained supervisors to maintain the data quality. Questionnaire-based data collectors were not informed about the scabies status of children. The questionnaire was piloted on 5% of the sample size in a rural *kebele* near the district. The collected data were checked by the supervisors and the principal investigators for completeness daily. First, the questionnaire was developed in English and then translated into the Amharic language. After data collection, the open responses of the study subjects were translated into English by experienced language translators.

### Variables and measurements

The variables of this study were operationally defined as follows:

A confirmed scabies case is the presence of clinical signs and symptoms of the disease in a child with mites, eggs or faeces on a skin scraping sample through light microscopy (Level A1) [32, 33].

Infestation is the presence of an unusually large number of insects or animals in a place, typically causing damage or disease.

Average daily water consumption is the average water usage of a person per day with a consumption of more than 20 L/capita/day (L/C/D) measured as adequate water quantity.

HH solid waste disposal is the removal of solid waste in a disposal pit, composting in a compound or burning in a pit.

HH liquid waste management is the use of septic tanks or soak pits in the compound to properly discharge the liquid wastes.

Frequent cloth washing is the washing of clothes two to four times a month.

Frequent bathing means taking a shower four to five times a month.

Caregivers’ knowledge about scabies includes information about its causative agent, signs and symptoms, commonly affected body parts, methods of transmission, vulnerable population groups, disease outcomes, and prevention methods. For this reason, a composite variable, the knowledge score, was computed by summing the subvariables. Then, since the mean value and median value of the score were similar, the mean value [11] was used as a cut-off value to classify the caregiver’s knowledge about scabies. Caregivers who scored more than the mean value were considered to have high knowledge, and caregivers who scored the mean or below were considered to have low knowledge.

Numerous subvariables, including binary and ordinal variables, were used to develop the HH wealth index for rural Ethiopia. The subvariables were adapted from various studies [34] and the Ethiopian Demographic and Health Survey [35]. The subvariables used for HH wealth index construction were longer-run and shorter-run HH assets, housing structure, farmland size, domestic animal size, mills, carts, generators for irrigation purposes, and beekeeping. Value weights for the variables ranged from 0 to 1, standing for the lowest and the highest values, respectively.

### Data analysis

The collected data were checked, double entered, coded, cleaned and verified using *EpiData Version 3.1* software (EpiData Association, Odense, Denmark). Statistical analysis was performed in STATA version 14.0 (Statistical Software: College Station, TX 77845, USA). However, SPSS statistical software version 24 (IBM SPSS Statistics for Windows; NY, USA) was used for principal component analysis (PCA). For continuous variables, the mean ± SD (standard deviation) or median with IQR (interquartile range) was calculated depending on the distribution of the data. For categorical variables, frequencies (*n*) and percentages (%) were calculated.

HH wealth was estimated by PCA [34]. Ranking of HHs into three groups was performed by considering Principal component 1. Then, cut-off values were set for the three groups. Standardization was carried out for the subvariables of the HH wealth index. A third variable was formed for each standardized variable from the product of the transformed variable and principal component 1. Finally, the third variables were summed, and a composite variable, the HH wealth score, was derived. A final categorical variable, the HH wealth index, was formed based on the cut-off values of principal component 1 ranks. It was classified into tertiles, with the higher tertile signifying higher wealth.

Bivariable and multivariable binary logistic regressions were modelled to identify independent factors associated with scabies infestation. An unadjusted analysis was run, and variables with *P* < 0.1 were modelled in the adjusted analysis. In the multivariable adjustment, adjusted odds ratios (AORs) with 95% confidence intervals (CIs) and *P* values are presented. Variables at *P* < 0.05 were reported as independent factors associated with scabies infestation. The Hosmer–Lemeshow goodness-of-fit test [36] with *P* > 0.05 was run to check the model fitness, which was *P* = 0.354.

Standard errors (SEs) and correlation matrices were used to check the collinearity of the associated variables. The maximum value of SE for the regression coefficient was 0.92, indicating no collinearity, since the value is in the range of − 2 < SE < 2. In addition, the variables had Pearson correlation values of less than 0.2 in the correlation matrix, signifying the absence of collinearity. Moreover, variance inflation factor (VIF) was calculated for each associated variable to check for multicollinearity with values below 10, an indicator of no multicollinearity.

## Results

### Sociodemographic characteristics of children and caregivers

Nine hundred forty-two children aged below 15 years participated in the study, with a 97.4% response rate. Nonresponse occurred due to refusal of HHs to be enrolled, opposition of a child to give a skin scarping sample, or absence of a child or mother/caregiver during revisiting sessions. The mean (SD) age of the caregivers was 37.2 (± 7.7) years. The mean (SD) age of children aged below 15 years was 6.8 (± 3.6) years. The median (IQR) family size was 5 [4–6] members in the HH. The median (IQR) number of beds was 1 [1, 2] in the HH. Out of 942 children, 492 (52.2%) were females. Five hundred seventy-two (60.7%) children lived in poor HHs (Table 1).

**Table 1:** Sociodemographic characteristics of children and caregivers in rural villages of *Lay Gayent District*, northwest Ethiopia, February–April 2021

Variables	Scabies infestation		
	Yes *n* (%)	No *n* (%)	Total *n* (%)
Caregiver sex			
Male	1 (0.5)	1 (0.1)	2 (0.2)
Female	202 (99.5)	738 (99.9)	940 (99.8)
Caregiver age (years)			
≤ 40	132 (65.0)	490 (66.3)	622 (66.0)
> 40	71 (35.0)	249 (33.69)	320 (34.0)
Religion			
Christian	187 (92.1)	677 (91.6)	864 (91.7)
Muslim	16 (7.9)	62 (8.4)	78 (8.3)
Maternal marital status			
Single	1 (0.5)	3 (0.4)	4 (0.4)
Married	199 (98.0)	720 (97.4)	919 (97.6)
Divorced	2 (1.0)	14 (1.9)	16 (1.7)
Widowed	1 (0.5)	2 (0.3)	3 (0.3)
Maternal education			
Unable to read and write	65 (32.0)	190 (25.7)	255 (27.1)
Able to read and write (non formal)	62 (30.5)	251 (34.0)	313 (33.2)
Grade 1–6 completed	48 (23.7)	198 (26.8)	246 (26.1)
Grade 7–12 completed	26 (12.8)	91 (12.3)	117 (12.4)
Above grade 12	2 (1.0)	9 (1.2)	11 (1.2)
Maternal occupation			
Housewife	174 (85.7)	662 (89.6)	836 (88.8)
Farmer	7 (3.5)	17 (2.3)	24 (2.6)
Merchant	2 (1.0)	15 (2.0)	17 (1.8)
Government employee	18 (8.8)	45 (6.1)	63 (6.6)
Day laborer	2 (1.0)	0	2 (0.2)
Paternal education			
Unable to read and write	39 (19.2)	128 (17.4)	167 (17.7)
Able to read and write (nonformal)	78 (38.4)	281 (38.0)	359 (38.1)
Grade 1–6 completed	64 (31.5)	218 (29.5)	282 (29.9)
Grade 7–12 completed	20 (9.9)	103 (13.9)	123 (13.1)
Above grade 12	2 (1.0)	9 (1.2)	11 (1.2)
Paternal occupation			
Farmer	182 (89.7)	640 (86.6)	822 (87.3)
Merchant	8 (3.9)	34 (4.6)	42 (4.5)
Government employee	11 (5.4)	51 (6.9)	62 (6.5)
Day laborer	2 (1.0)	14 (1.9)	16 (1.7)
Family size			
2–5 persons	129 (63.6)	512 (69.3)	641 (68.1)
> 5 persons	74 (36.4)	227 (30.7)	301 (31.9)
Number of bed(s)			
1–2	190 (93.6)	692 (93.6)	882 (93.6)
3–4	13 (6.4)	47 (6.4)	60 (6.4)
Child sex			
Male	96 (47.3)	354 (47.9)	450 (47.8)
Female	107 (52.7)	385 (52.1)	492 (52.2)
Child age (years)			
1–5	81 (39.9)	304 (41.1)	385 (40.9)
6–10	77 (37.9)	307 (41.6)	384 (40.7)
11–14	45 (22.2)	128 (17.3)	173 (18.4)
Number of under 15 child (ren)			
1–2	159 (78.3)	613 (82.9)	772 (81.9)
3–4	44 (21.7)	126 (17.1)	170 (18.1)
HH wealth status			
Poor	110 (54.2)	462 (62.5)	572 (60.7)
Medium	6 (3.0)	34 (4.6)	40 (4.3)
Rich	87 (42.8)	243 (32.9)	330 (35.0)

### Housing characteristics

Nearly all of the children, 937 (99.5%), had lived in a house with an earth/sand floor. Three-quarters of children, 700 (74.3%), had lived in a corrugated iron-roof house (Table 2).

**Table 2:** Housing characteristics of children in rural villages of *Lay Gayent District*, northwest Ethiopia, February–April 2021

Variables	Scabies infestation		
	Yes *n* (%)	No *n* (%)	Total *n* (%)
Roofing materials			
Thatch	66 (32.5)	176 (23.8)	242 (25.7)
Corrugated iron	137 (67.5)	563 (76.2)	700 (74.3)
Wall building materials			
Mud	203	735 (99.5)	938 (99.6)
Cement	0	4 (0.5)	4 (0.4)
Flooring materials			
Earth/sand	202 (99.5)	735 (99.5)	937 (99.5)
Cement	1 (0.5)	4 (0.5)	5 (0.5)
Having separate kitchen			
Yes	109 (53.7)	531 (71.9)	640 (67.9)
No	94 (46.3)	208 (28.1)	302 (32.1)
Tethering of cattle, goats and sheep^a^			
No	58 (28.6)	255 (34.5)	313 (33.2)
Yes	145 (71.4)	484 (65.5)	629 (66.8)
Number of rooms in the house			
1	55 (27.1)	153 (20.7)	208 (22.1)
2	53 (26.1)	199 (26.9)	252 (26.8)
3	95 (46.8)	387 (52.4)	482 (51.1)

^a^Tethering indoors at night before the survey

### Water supply and sanitation characteristics of households

More than two-thirds of the HHs, 633 (67.2%), faced water supply interruptions. A large proportion of HHs, 571 (60.6%), had cleaned their house every day. In addition, a considerable proportion of HHs, 580 (61.6%), openly disposed of their solid wastes into the surrounding environment (Table 3).

**Table 3:** Water supply and sanitation characteristics of HHs in rural villages of *Lay Gayent District*, northwest Ethiopia, February–April 2021

Variables	Scabies infestation		
	Yes *n* (%)	No *n* (%)	Total *n* (%)
Water source type			
Public tap	56 (27.6)	267 (36.1)	323 (34.3)
Piped to yard/compound	2 (1.0)	2 (0.3)	4 (0.4)
Protected hand-dug well	27 (13.3)	132 (17.8)	159 (16.9)
Protected spring	19 (9.4)	96 (13.0)	115 (12.2)
Unprotected spring	22 (10.8)	67 (9.0)	89 (9.5)
Unprotected hand-dug well	77 (37.9)	175 (23.8)	252 (26.7)
Round-trip water collection time^a^			
1–30 min	133 (65.5)	518 (70.1)	651 (69.1)
> 30 min	70 (34.5)	221 (29.9)	291 (30.9)
Daily water consumption (average)			
≤ 20 L/C/D	37 (18.2)	90 (12.2)	127 (13.5)
> 20 L/C/D	166 (81.8)	649 (87.8)	815 (86.5)
Water supply interruption			
No	74 (36.5)	235 (31.8)	309 (32.8)
Yes	129 (63.5)	504 (68.2)	633 (67.2)
Availability of functional latrine			
No	52 (25.6)	138 (18.7)	190 (20.2)
Yes	151 (74.4)	601 (81.3)	752 (79.8)
Daily house cleaning			
No	111 (54.7)	260 (35.2)	371 (39.4)
Yes	92 (45.3)	479 (64.8)	571 (60.6)
HH liquid waste disposal			
Proper discharging^b^	30 (14.8)	165 (22.3)	195 (20.7)
Open discharging	173 (85.2)	574 (77.7)	747 (79.3)
Solid waste disposal			
Proper^c^	59 (29.1)	303 (41.0)	362 (38.4)
Openly into the field	144 (70.9)	436 (59.0)	580 (61.6)

^a^Self-reported by water collectors

^b^Using a soak pit or septic tank

^c^Burning, composting, or dumping in a pit

### Personal hygiene of children and knowledge of caregivers about scabies

Most of the children under 15 years of age, 839 (89.1%), had washed their clothes 2–4 times in a month by themselves or through their caregivers. Ninety-two (9.8%) of the children had shared clothes with a scabies case. The majority of child caregivers, 533 (56.6%) had low levels of knowledge about scabies (Table 4).

**Table 4:** Personal hygiene of children and caregivers’ knowledge about scabies in rural villages of *Lay Gayent District*, northwest Ethiopia, February–April 2021

Variables	Scabies infestation		
	Yes *n* (%)	No *n* (%)	Total *n* (%)
Cloth washing frequency			
2–4 times per month	190 (93.6)	649 (87.8)	839 (89.1)
Once per month	13 (6.4)	90 (12.2)	103 (10.9)
Bathing frequency			
4–5 times per month	36 (17.7)	71 (9.6)	107 (11.4)
1–3 times per month	167 (82.3)	668 (90.4)	835 (88.6)
Using soap or *Endod* for bathing^a^			
Yes	43 (21.2)	147 (19.9)	190 (20.2)
No	160 (78.8)	592 (80.1)	752 (79.8)
Trimming of nails			
Yes	66 (32.5)	431 (58.3)	497 (52.8)
No	137 (67.5)	308 (41.7)	445 (47.2)
Sharing clothes with a scabies case^b^			
No	136 (67.0)	714 (96.6)	850 (90.2)
Yes	67 (33.0)	25 (3.4)	92 (9.8)
Using soap for hand washing			
Yes	17 (8.4)	117 (15.8)	134 (14.2)
No	186 (91.6)	622 (84.2)	808 (85.8)
Caregiver knowledge about scabies			
High knowledge	50 (24.6)	359 (48.6)	409 (43.4)
Low knowledge	153 (75.4)	380 (51.4)	533 (56.6)

^a^ soap berry (*Endod*: *Phytolacca dodecandra*) used for washing clothes or bathing in rural and peri-urban areas

^b^ Sharing clothes for a prolonged time with a suspected or confirmed case in the 2 months prior to the survey

### Scabies infestation and associated independent factors

The prevalence of scabies infestation among children aged below 15 years in rural villages of *Lay Gayent District* was 21.5% (95% CI 19.0–24.3). In the multivariable analysis, illiteracy of mothers (AOR = 1.61; 95% CI 1.07–2.43); poor HH wealth status (AOR = 2.04; 95% CI 1.25–3.33); unimproved water source (AOR = 1.58; 95% CI 1.05–2.40); not cleaning of a house daily (AOR = 2.43; 95% CI 1.63–3.62); not trimming of child nails (AOR = 2.21; 95% CI 1.50–3.25); child cloth sharing with a scabies case (AOR = 11.77; 95% CI 6.94–19.97); and low caregiver knowledge about scabies (AOR = 2.44; 95% CI 1.64–3.63) were independent factors associated with scabies infestation (Table 5).

**Table 5:** Independent factors associated with scabies infestation in rural villages of *Lay Gayent District*, northwest Ethiopia, February–April 2021

Variables	COR (95% CI)	*P* value	AOR (95% CI)	*P* value
Maternal education				
Able to read and write	1		1	
Grade 7–12 completed	1.17 (0.72–1.89)	0.533	1.28 (0.73–2.24)	0.394
> 12	0.91 (0.19–4.26)	0.902	0.99 (0.16–6.02)	0.996
Unable to read and write	1.40 (0.98–1.98)	0.062	1.61 (1.07–2.43)	*0.023*
HH wealth status				
Rich	1		1	
Medium	0.49 (0.20–1.21)	0.124	1.44 (0.52–4.00)	0.488
Poor	0.67 (0.48–0.92)	0.013	2.04 (1.25–3.33)	*0.004*
Water source type				
Improved source	1	< 0.001	1	
Unimproved source	1.95 (1.43–2.68)		1.58 (1.05–2.40)	*0.030*
Daily water consumption (average)				
> 20 L/C/D	1	0.026	1	0.449
≤ 20 L/C/D	1.61 (1.06–2.44)		1.21 (0.74–1.99)	
Daily house cleaning				
Yes	1	< 0.001	1	< *0.001*
No	2.22 (1.62–3.01)		2.43 (1.63–3.62)	
HH liquid waste disposal				
Proper discharging	1	0.020	1	0.100
Open discharging	1.66 (1.08–2.53)		1.56 (0.92–2.66)	
Solid waste disposal				
Proper	1	0.002	1	0.185
Open	1.70 (1.21–2.37)		1.32 (0.88–1.99)	
Cloth washing frequency				
2–4 times per month	1		1	0.246
Once per month	0.49 (0.27–0.90)	0.022	0.66 (0.33–1.33)	
Bathing frequency				
4–5 times per month	1	0.001	1	0.066
1–3 times per month	0.49 (0.32–0.76)		0.61 (0.36–1.03)	
Trimming of nails				
Yes	1	< 0.001	1	< *0.001*
No	2.9 (2.09–4.03)		2.21 (1.50–3.25)	
Sharing clothes with scabies case				
No	1	< 0.001	1	< *0.001*
Yes	14.07(8.58–23.07)		11.77 (6.94–19.97)	
Using soa*P* for hand washing				
Yes	1	0.008	1	0.125
No	2.06 (1.21–3.51)	0.008	1.62 (0.87–3.02)	
Caregiver knowledge about scabies				
High knowledge	1	< 0.001	1	< *0.001*
Low knowledge	2.89 (2.04–4.10)		2.44 (1.64–3.63)	

The italics re*P*resent statistical significance during multivariable adjustment (*P* < 0.05); COR: crude odds ratio

## Discussion

In this study, a 21.5% prevalence of scabies was determined among children aged below 15 years in rural villages of *Lay Gayent District*, northern Ethiopia. This prevalence is higher than that in studies conducted among schoolchildren in southern Ethiopia: 5.5% by Walker et al. [4], 5.3% by Amare and Lindtjorn [22], and 17% among rural children by Figueroa et al. [37]; in northern Ethiopia, 9.3% by Dagne et al. [24], and in northwest Ethiopia, 10.8% among school-age children by Misganaw et al. [20]. However, this finding is similar to that of a meta-analysis performed in Ethiopia among children (19.5%) [19]. Moreover, other studies performed elsewhere in the world had a lower prevalence among schoolchildren or school-aged children: 10.2% in India [38], 18.5% in Fiji [39], 8.1% among students in Malaysian secondary boarding schools [40], 4.4% in Egypt [21], 17.8% among Cameroonian boarding schools [41], 5.2% in the dry season and 1.9% in the wet season in Guinea-Bissau [17], and 3.1% in southeastern Iran [18]. In contrast, this finding is lower than results from other studies: 97.8% among children in Islamic education institutes in Dhaka, Bangladesh [23], 31% among children in Malaysian welfare homes [42], 55.7% in children aged 5–9 years in Fiji [43], and 25.7% in children aged 1–4 years in the Solomon Islands [16]. These differences could occur due to variability in demography, socioeconomics, societal culture, study population, study setting, individual hygiene, domestic/housing hygiene, behaviors, including sharing of clothes or other objects with a scabies case, environmental hygiene, climatic condition/weather, or nature of disease occurrence.

Maternal illiteracy was a significant factor associated with scabies infestation among children. This information is similar to the findings of previous studies in Nigeria [10], Egypt [21], Iran [18], and Dhaka, Bangladesh [23]. In rural Ethiopian communities, mothers are the most common primary caregivers for their children, which is critically important for the prevention of communicable diseases and child health. Thus, the possible explanation for this finding is that illiterate mothers are less informed about the nature of the disease despite the information being available through face-to-face health education and delivery of printed manuals by health extension programs. As a result, having no information about its transmission and prevention methods may prevent them from being protected.

Living in a poor HH had children who were heavily exposed to scabies in rural communities. Similar findings are documented in prior studies [10, 18, 23]. This might be because poor HHs are characterized by poor housing conditions, inadequate domestic hygiene, poor personal hygiene, illiteracy, small housing size, busy work schedules, a lack of time to maintain individual hygiene, and the unavailability of soap/detergents to maintain personal hygiene, which predispose individuals to be infested by itch mites.

Households that obtained water from unimproved sources had higher odds of scabies infestation for their children. It has been documented that unimproved water sources or inadequate water supplies produce considerable scabies burdens in low- and middle-income countries [44]. This finding may be linked with the inaccessibility of water sources due to the long water collection time, unpleasant nature of water sources, intermittent water supply or water scarcity, poor socioeconomic status of hamlets, and inequitable distribution of improved water sources by the village administration. These factors may in turn lead to poor personal hygiene, which is a direct explanatory variable of scabies infestation. A community-based study conducted in drought-stricken areas of Ethiopia reported the highest prevalence of scabies [3].

Households that had not cleaned a house daily had increased odds of having a child with scabies infestation. This finding is aligned with a study in Islamic education institutes in Dhaka, Bangladesh [23]. In rural Ethiopia, persons, mainly children, usually sleep on a plinth or a long chair placed on the floor or sometimes even on the floor, which can harbor itch mites for several days. In addition, HH members regularly sit on a plinth or chair for dinner with subsequent long talk or coffee drinking. It is widely reported that crusted (Norwegian) scabies can also be transmitted by exposure to contaminated objects, including furniture and the housing itself [45, 46]. Therefore, the findings of this study could be explained by the type of sleeping arrangement for a child, the clinical type of scabies, and whether the child lapses his or her daily time in the house.

Nail hygiene is important in preventing the transmission of infections. Many disease-causing organisms can be harbored in fingernails, resulting in disease spread [47, 48]. It was found that children with untrimmed fingernails were more likely to be infested with scabies mites than children with trimmed fingernails. Consistent findings were observed in previous studies [22]. This might result from poor child healthcare by parents or caregivers, unavailability of razor blades or unaffordability of nail cutters, difficulty of avoiding mites inside the nails while washing hands even with sanitizers or detergents, or obtaining thin or smooth skin surfaces for mites at the proximal part of the nails.

Sharing clothes with a scabies case was also associated with increased odds of risk of scabies. This finding is in line with findings of other studies [10, 11, 18, 21]. This is highly likely because the presence of scabies mites on the clothes of a patient for several days assures prolonged contact to ease transmission of the mite to a healthy person. Cloth sharing may exist due to poor socioeconomic status, the absence of washed or clean clothes for change, and the cultural value of rural communities, which are characteristics of this study population.

Caregivers or mothers who had low knowledge about scabies made children more prone to scabies infestation. Studies have also documented the impact of poor behavior on seeking health care, which results in untreated and severe morbidity [6]. The findings of this study can be explained by the poor awareness of caregivers or mothers about the transmission and prevention methods of scabies, which may result in poor practice of its prevention methods. Furthermore, this may make their children more vulnerable to scabies infestation. Numerous studies have shown the significant contribution of insufficient application of prevention methods to prevent infestation by scabies mites [10, 19, 20, 22, 24].

This study used the robust, community-based cluster random sampling approach. However, to date, the majority of similar studies have used epidemiologically less sound methods, possibly leading to incorrect prevalence estimates. This study addressed all age groups of children who are more vulnerable to scabies. A large representative sample of rural children was used in this study, making the results inferable to rural children in comparable socioeconomic and cultural settings.

This survey had some methodological problems. The causal relationship between the exposures and scabies infestation cannot be concluded with certainty. The exclusion of asymptomatic mite carriers may influence the prevalence of scabies. Self-reported measurement errors on exposure variables, round-trip water collection time and average daily water consumption may be introduced, since the caregivers in rural areas are mainly illiterate. Certain information related to individual hygiene and domestic hygiene may be influenced by self-reporting bias or social desirability bias. Although unmeasured variables were not controlled, the influence on the findings is limited.

## Conclusion and recommendation

Scabies remains a significant public health issue among children aged below 15 years in rural areas of the district and is similar to the national average (19.5%). Maternal illiteracy, low HH wealth, unimproved water source, not cleaning of a house daily, not trimming of child nails, child cloth sharing with a scabies case, and low caregiver knowledge about scabies were factors associated with scabies infestation.

Thus, this study recommends improving mothers’ education through various approaches in collaboration with the District Education Office and caregivers’ knowledge about scabies via information, education and communication, taking their educational status into account. In addition, it is recommended to upgrade HH wealth through microinvestments. In rural communities, a safe water supply, healthy housing, and adequate personal hygiene, including trimming of child nails and avoidance of child cloth sharing with a scabies case, should also be maintained optimum.

Longitudinal studies, particularly repeated cross-sectional studies involving all seasons of a year and ecoclimatic zones, are suggested to reach a better estimate of scabies prevalence in rural areas of Ethiopia, which helps to direct scabies-specific interventions.

## Data Availability

Data will be accessible from the corresponding author upon request.

## Abbreviations

AOR: Adjusted odds ratio
CI: Confidence interval
DALYs: Disability-adjusted life-years
Deff: Design effect
HH: Household
IQR: Interquartile range
L/C/D: Liters/capita/day
NTDs: Neglected tropical diseases
PCA: Principal components analysis
SD: Standard deviation
SE: Standard error
VIF: Variance inflation factor
WHO: World Health Organization
